# Perioperative Anesthetic Considerations for Temporal Bone Tumor Resection in Fibroblast Growth Factor 23 (FGF23)-Related Tumor-Induced Osteomalacia: A Case Report

**DOI:** 10.7759/cureus.116044

**Published:** 2026-09-10

**Authors:** Marcos R Latimer-Fantauzzi, Harold L Rivera-Alvarado, Hector Torres, Maria J Crespo

**Affiliations:** 1 Anesthesiology, University of Puerto Rico, Medical Sciences Campus, San Juan, PRI; 2 Anesthesiology, University of Puerto Rico School of Medicine, San Juan, PRI; 3 Physiology and Anesthesiology, University of Puerto Rico, Medical Sciences Campus, San Juan, PRI

**Keywords:** cranial neurosurgery, general anesthesia, phosphaturic mesenchymal tumors (pmts), tumor-induced osteomalacia (tio), volatile anesthesia

## Abstract

Phosphaturic mesenchymal tumors (PMTs) are uncommon, predominantly benign neoplasms frequently associated with dysregulated phosphate metabolism through the overproduction of fibroblast growth factor 23 (FGF23). Elevated levels of FGF23 lead to phosphate depletion, which may impair tissue oxygen delivery, central respiratory drive, and respiratory muscle function. Systemic manifestations may include osteomalacia and neuromuscular dysfunction, with an increased risk of respiratory and cardiovascular complications. PMTs often present with nonspecific clinical findings and may remain unrecognized during the perioperative period, presenting significant challenges for anesthetic planning and management. We report the case of a 72-year-old male with tumor-induced osteomalacia (TIO), a right temporal mass, and multiple comorbidities, including hypertension and obstructive sleep apnea. The patient reported progressive headaches and general weakness. After neurosurgical evaluation, he underwent elective surgical excision under general anesthesia. Intraoperatively, he developed hypotension following induction of anesthesia, requiring vasopressor support with ephedrine boluses and a continuous phenylephrine infusion. The remainder of the procedure was uneventful, and the patient was extubated and transferred to the intensive care unit in stable condition. This case highlights the importance of recognizing potential complications in patients with metabolic abnormalities presenting with PMTs, particularly disturbances in phosphate homeostasis that may have significant anesthetic implications. The use of video laryngoscopy, judicious administration of neuromuscular blocking agents, and an individualized anesthetic strategy facilitated secure airway management, minimized cervical manipulation, and supported an uneventful recovery. Moreover, integrating anesthetic selection with comprehensive perioperative planning is essential to address the metabolic, musculoskeletal, respiratory, and hemodynamic challenges associated with TIO, particularly in patients with significant comorbidities.

## Introduction

Phosphaturic mesenchymal tumors (PMTs) are rare mesenchymal neoplasms associated with tumor-induced osteomalacia (TIO) [1]. These tumors predominantly affect adults, with a mean age at diagnosis in the mid-to-late 40s and a slight male predominance [2]. More than 98% of PMTs are benign, relatively small (mean size approximately 3 cm), and most frequently located in the lower extremities in nearly half of cases and in the head and neck in approximately one-quarter [1-3].

Overproduction of fibroblast growth factor 23 (FGF23), a bone-derived protein produced by PMTs, is the primary underlying mechanism of TIO [2]. FGF23 plays a central role in phosphate homeostasis through its renal effects, including decreased phosphate reabsorption and suppression of vitamin D synthesis [4]. Dysregulation of FGF23, particularly in states of excess secretion, leads to hypophosphatemia and impaired skeletal mineralization, resulting in significant metabolic and musculoskeletal complications [1,4]. Phosphate depletion impairs adenosine triphosphate (ATP) synthesis and reduces intracellular 2,3-diphosphoglycerate levels, thereby affecting tissue oxygen delivery, central respiratory drive, and respiratory muscle function [5]. These alterations may contribute to respiratory muscle weakness, impaired ventilatory reserve, and increased susceptibility to perioperative respiratory complications. In addition, severe hypophosphatemia can cause generalized neuromuscular weakness and may contribute to delayed recovery from anesthesia and prolonged neuromuscular blockade. The impaired skeletal mineralization associated with TIO also results in substantial skeletal fragility, increasing the risk of pathologic fractures and positioning-related skeletal injury during the perioperative period [6].

Patients with TIO exhibit nonspecific symptoms, including bone pain, reported in up to 90-97% of cases, muscle weakness in 53-83%, and pathologic fractures in 69-85% [1,2,6]. Despite advances in functional imaging modalities, including somatostatin receptor-based PET/CT, delayed diagnosis remains common in patients with PMTs, with a mean time to diagnosis of approximately 4.8 years [1,2]. Although medical management may alleviate symptoms and partially correct hypophosphatemia [1,7], complete surgical resection remains the cornerstone of treatment, reducing morbidity and achieving clinical improvement in more than 95% of cases [1,2]. Despite the effectiveness of surgical resection, the literature on the perioperative management of patients with TIO remains limited, and evidence-based anesthetic guidelines have not been established. Current clinical and anesthetic considerations are largely derived from case reports and are primarily focused on tumor location, patient positioning, and strategies to mitigate respiratory compromise [1,2,6]. Conditions such as obstructive sleep apnea (OSA) and hypertension are known to make perioperative airway management and hemodynamic stabilization much more complex [8,9]. In the setting of TIO, these comorbidities create additional perioperative challenges requiring a tailored anesthetic approach. Thus, the anesthetic management of patients with TIO and hypophosphatemia requires particular attention to respiratory and neuromuscular function, skeletal protection, and the prevention and recognition of perioperative complications. The limited literature addressing these specific anesthetic considerations highlights an important knowledge gap that this case seeks to address. We report the successful anesthetic management of a patient with TIO, OSA, and hypertension who underwent resection of a cranial PMT under general anesthesia, emphasizing the strategies used to address the patient’s metabolic, airway, and hemodynamic risks.

## Case presentation

A 72-year-old man with a past medical history significant for hypertension, OSA, migraines, benign prostatic hyperplasia, and TIO presented to the emergency department with severe headache, blurred vision, and progressive upper and lower extremity weakness. On admission, the patient reported localized pain at the right temporal site, first noticed one year ago.

His past medical history included a nontraumatic left femur fracture requiring surgical repair, a right total knee arthroplasty, and an appendectomy. Previous MRI findings revealed diffuse fractures affecting the bilateral proximal femurs, right hip fracture, bilateral iliac wings, right inferior pubic ramus, right lesser trochanter, bilateral scapulae, right glenoid, and multiple bilateral ribs, as well as chronic compression fractures of L2 and L4. Several fractures demonstrated osteolytic and pathologic characteristics. Ten months prior to the current procedure, FGF23 levels were found to be markedly elevated at 756 RU/mL (normal <180 RU/mL), raising suspicion of a PMT. A 68Ga-DOTATATE PET/CT confirmed a somatostatin receptor-positive right temporoparietal scalp lesion superficial to the temporal bone measuring approximately 1.2 cm in depth, with a markedly elevated standardized uptake value of 71.5, highly suggestive of a PMT.

Home medications included amlodipine (5 mg PO, daily), doxepin (150 mg PO, daily), finasteride (5 mg PO, daily), pregabalin (300 mg PO, daily), sulindac (150 mg PO, daily), and tamsulosin (0.4 mg PO, daily), in addition to supplementation with calcitriol (0.5 mcg PO, BID) and potassium phosphate/sodium phosphate (250 mg PO, daily). The patient reported a penicillin allergy. He was a former smoker, having quit 30 years prior, and denied alcohol or illicit drug use. On physical examination, vital signs were within normal limits. Neurologic examination was notable for decreased muscle strength graded 4/5 in both the upper and lower extremities. On evaluation, the patient was noted to have limited mobility, requiring assistance with ambulation and spending most of the time on bed rest.

The remainder of the physical examination was unremarkable except for extreme tenderness over the right temporal region, rated at 10/10 on the Numeric Rating Scale. CT imaging showed a well-circumscribed, low-density mass measuring up to 1.9 cm in maximal diameter, located within the posterosuperior aspect of the right temporalis muscle (Figure 1). Considering the progressive neurologic symptoms and imaging findings, the patient was admitted, and the case was discussed with the neurosurgery service, which recommended surgical intervention following evaluation. The patient was subsequently scheduled for mass excision and right temporal bone resection the following day. The anesthesiology service was consulted for perioperative evaluation and anesthetic planning.

**Figure 1:**
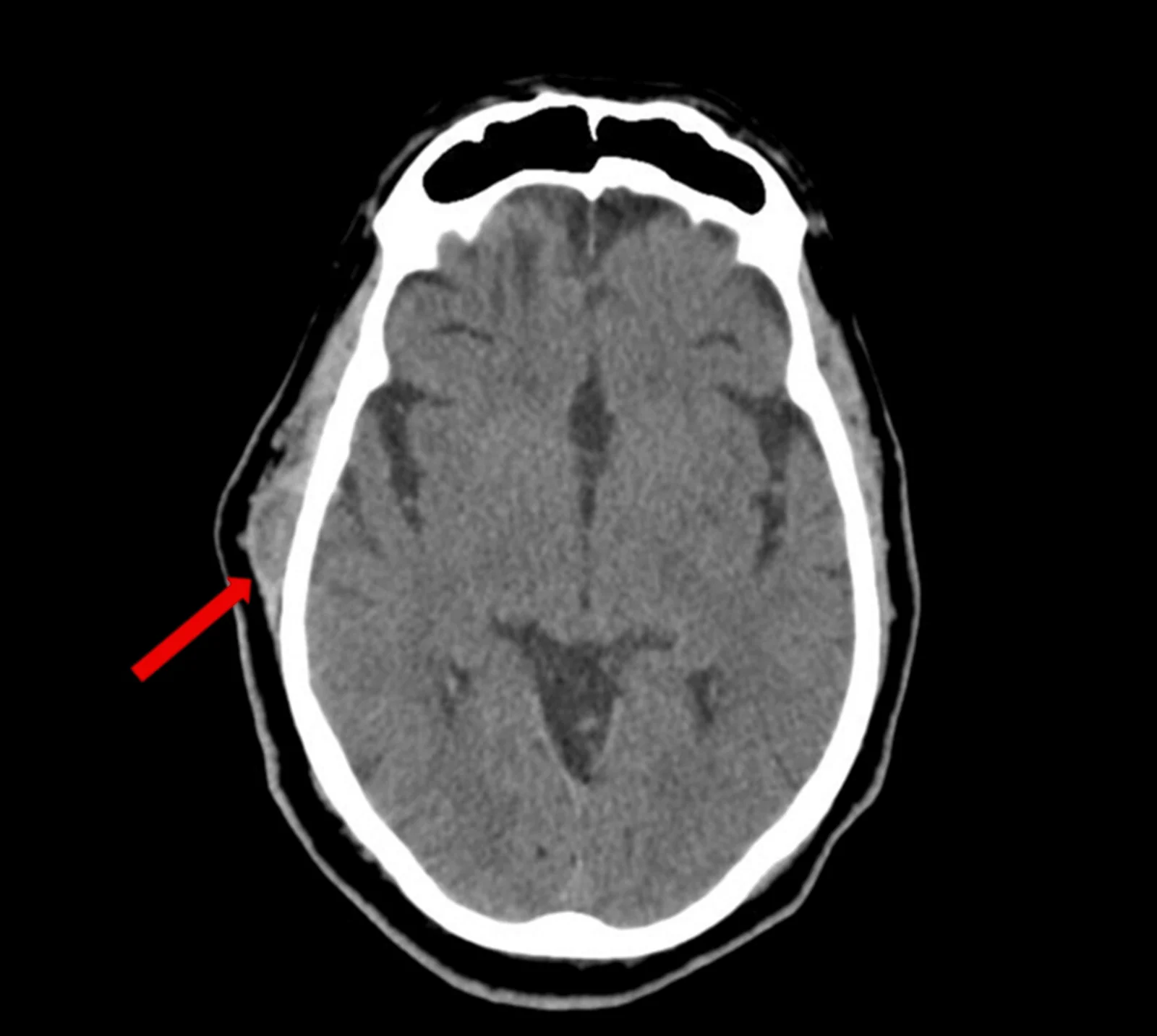
Preoperative CT image (axial view) of the skull. The red arrow indicates a low-density mass occupying the right posterosuperior aspect of the temporalis muscle, measuring approximately 1.9 cm in diameter.

During anesthetic preoperative evaluation, the patient was alert and oriented to person, time, and place. He fasted for over eight hours prior to the procedure. The patient weighed 86 kg and was 175.3 cm tall. His vital signs included an elevated blood pressure (BP) of 141/99 mmHg, a heart rate (HR) of 103 bpm, a respiratory rate (RR) of 20 bpm, and a peripheral oxygen saturation (SpO₂) of 92% on room air. Preoperative laboratory results are shown in Table 1. On evaluation, the patient showed lower extremity weakness and complained of headache, blurry vision, and generalized facial pain. Airway examination revealed a Mallampati class III with normal dentition, mouth opening, and neck range of motion. The patient was classified as American Society of Anesthesiologists (ASA) physical status III [10]. A 20-gauge peripheral IV (IV) catheter was placed in the left arm. He was subsequently transported to the operating room, arriving in calm condition.

**Table 1:** Preoperative laboratory results.

Test	Result	Normal range
Complete blood count		
White blood cell count (× 10³/µL)	4.99	3.98-10.04
Red blood cell count (× 10⁶/µL)	4.94	4.63-6.08
Hemoglobin (g/dL)	15	13.7-17.5
Hematocrit (%)	46.2	40.1-51.0
Platelets (× 10³/µL)	137	163.0-369.0
Coagulation panel		
Prothrombin time (seconds)	11.6	9.00-11.50
Partial thromboplastin time (seconds)	37.6	22.2-34.0
International normalized ratio	1.05	0.9-1.1
Complete metabolic panel		
Sodium (mmol/L)	143	135.0-145.0
Potassium (mmol/L)	4	3.5-5.1
Chloride (mmol/L)	99	98.0-107.0
Carbon dioxide, total (mmol/L)	32.9	22.0-29.0
Blood urea nitrogen (mg/dL)	23.3	8.0-23.0
Glucose (mg/dL)	119	70.0-99.0

After arrival at the operating room, the patient was carefully moved to the operating table in a supine position. Foam supports and blankets were placed over specific pressure points, and ASA standard monitors were promptly connected. The patient was premedicated with midazolam (2.5 mg, IV) for anxiolysis, and clindamycin (480 mg, IV) was administered at the request of the neurosurgical team. A facial mask with 100% oxygen was placed in preparation for rapid-sequence induction of anesthesia. For induction, propofol (180 mg, IV), fentanyl (100 mcg, IV), lidocaine (100 mg, IV), succinylcholine (80 mg, IV), and rocuronium (10 mg, IV) were used. Successful intubation was achieved on the first attempt using a McGRATH™ MAC3 video laryngoscope (Medtronic, Minneapolis, MN, USA) with a 7.5-mm endotracheal tube (ETT), and dexamethasone (4 mg, IV) was administered shortly after. Mechanical ventilation settings were adjusted taking into consideration the patient’s underlying OSA. Pressure-controlled ventilation-volume guaranteed (PCV-VG) mode was used with the following parameters: RR of 16 breaths/min, tidal volume of 500 mL, positive end-expiratory pressure of 5 cmH₂O, and fraction of inspired oxygen of 55%. In addition, an 18-gauge peripheral IV catheter was placed in the right arm to provide rapid and reliable venous access in the event of hemodynamic instability. Anesthesia maintenance was achieved with sevoflurane at 1-1.5%, reaching 0.5-0.7 MAC, and muscle paralysis was continued with an additional dose of rocuronium (40 mg, IV).

BP was monitored using serial noninvasive measurements. Before induction, his BP was 145/90 mmHg. Following induction, the patient developed hypotension, with BP decreasing from 130/70 mmHg (mean arterial pressure (MAP), 90 mmHg) to 85/60 mmHg (MAP, 68 mmHg). Two IV boluses of ephedrine (10 mg each) were administered along with two 500-mL boluses of IV 0.9% normal saline solution. Additionally, a continuous IV infusion of phenylephrine was initiated at 30 mcg/min and titrated to 70 mcg/min until hemodynamic stability was achieved. The MAP remained below the predefined target of 70 mmHg for approximately 10 minutes. Intraoperatively, systolic BP ranged from 85 to 108 mmHg and diastolic BP from 60 to 68 mmHg, with a MAP maintained between 68 and 76 mmHg. The patient’s temporal cranial mass was successfully excised with an approximate 1-cm margin of surrounding tissue to ensure complete resection. A local infiltration of 1% xylocaine with epinephrine (1:100,000) was administered at the incision site.

The procedure started approximately 20 minutes after induction and lasted approximately one hour and five minutes, with an estimated blood loss of 50 mL. A total of 2,000 mL of 0.9% normal saline was administered, with a urine output of 150 mL (0.9 mL/kg/h), resulting in a net positive fluid balance of 1,850 mL. Despite hypotension during induction, the procedure was well tolerated by the patient, without ventilation or respiratory difficulties, electrocardiogram abnormalities, or additional complications. Following closure of the wound site, the vasopressor infusion was discontinued, and BP remained stable. Paralysis was reversed with sugammadex (200 mg, IV), and spontaneous ventilation was achieved. The ETT cuff was deflated, and the patient was successfully extubated and transferred to the post-anesthesia care unit (PACU) in stable condition. Vitals in the PACU showed BP of 99/66 mmHg, RR of 19 bpm, HR of 100 bpm, and SpO₂ of 98% on nasal cannula.

After stabilization, the patient was transferred to the neurosurgery intensive care unit for continued observation, with close monitoring of electrolytes and seizure prophylaxis. At evaluation on postoperative day 1, a Glasgow Coma Scale score of 15 with adequate surgical wound healing was observed. A brain MRI, along with routine laboratory tests including an intact parathyroid hormone profile, was ordered (Figure 2, Table 2).

**Figure 2:**
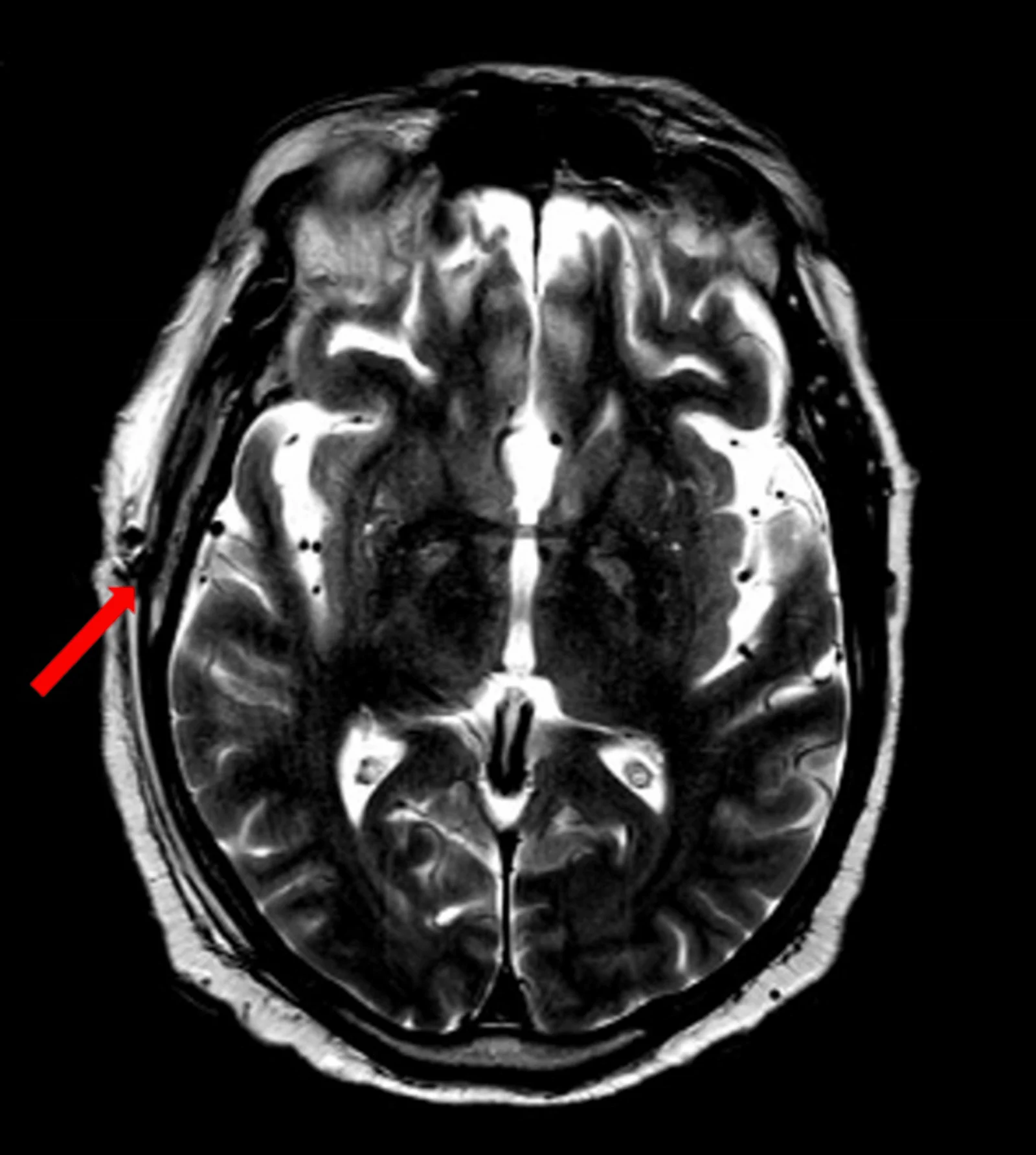
Postoperative day 2 MRI (axial view) following resection of the PMT. The red arrow indicates a small amount of subcutaneous edema and emphysema at the right temporal surgical site. PMT, phosphaturic mesenchymal tumor

**Table 2:** Postoperative laboratory results. ALT, alanine transaminase; AST, aspartate transaminase; PTH, parathyroid hormone; SGOT, serum glutamic-oxaloacetic transaminase; SGPT, serum glutamic-pyruvic transaminase

Test	Result	Normal range
Complete blood panel		
White blood count (× 10³/µL)	5.26	3.98-10.04
Red blood count (× 10⁶/µL)	4.86	4.63-6.08
Hemoglobin (g/dL)	14.7	13.7-17.5
Hematocrit (%)	45.6	40.1-51.0
Platelets (× 10³/µL)	128	163.0-369.0
Coagulation panel		
Prothrombin time (seconds)	11.4	9.00-11.50
Partial thromboplastin time (seconds)	35.6	22.2-34.0
International normalized ratio	1.03	0.9-1.1
Complete metabolic panel		
Sodium (mmol/L)	143	135.0-145.0
Potassium (mmol/L)	4.6	3.5-5.1
Chloride (mmol/L)	103	98.0-107.0
Carbon dioxide, total (mmol/L)	29	22.0-29.0
Blood urea nitrogen (mg/dL)	20.8	3.5-5.1
Creatinine (mg/dL)	1	0.5-1.5
Glucose (mg/dL)	130	70.0-99.0
Alkaline phosphatase (U/L)	230	40.0-129.0
SGPT (ALT) (U/L)	22	5.0-50.0
SGOT (AST) (U/L)	19	5.0-50.0
Albumin (g/dL)	4.1	3.5-5.0
Phosphorus (mg/dL)	2.3	2.5-5.0
Calcium (mg/dL)	9.1	8.2-10
Magnesium (mg/dL)	1.8	1.6-2.4
PTH, Intact (pg/dL)	32.6	12.0-88.0
Calcitriol (pg/mL)	37.6	19.90-79.30

The postoperative course was uneventful, with the patient demonstrating stable neurologic and hemodynamic status. Postoperatively, he received acetaminophen (1 g, IV) and morphine (2 mg, IV), achieving satisfactory pain control without respiratory compromise. He was discharged home on postoperative day 2. Pathologic evaluation of the right extracranial parietal lesion demonstrated a vascular tumor consistent with a PMT. Histologic examination revealed bland ovoid-to-spindle cells embedded within a myxoid, hyalinized, and vascularized stroma, with focal areas of amorphous, grungy calcifications. Immunohistochemical staining was positive for SATB2, SSTR2, ERG, and CD56 and negative for EMA, CD31, CD34, SMA, desmin, S100, PR, and STAT6. At two weeks post-discharge, serum calcium and phosphorus concentrations remained within normal limits despite cessation of supplementation after surgery (Table 3).

**Table 3:** Perioperative phosphate and calcium levels. * Seven months prior to the procedure, with oral supplementation with potassium phosphate/sodium phosphate (250 mg, PO, daily) and calcitriol (0.5 mcg, PO, BID).

Laboratory parameters	Normal values	Preoperative*	Postoperative day 1	Postoperative day 2	Postoperative day 14
Phosphate (mg/dL)	2.5-5.0	2.88	2.3	2.4	4.6
Calcium (mg/dL)	8.8-10.0	9	9.1	8.8	10.2

## Discussion

We present the case of a 72-year-old patient with TIO who underwent resection of a temporal bone PMT under general anesthesia. Patients with TIO present unique anesthetic challenges, largely attributable to chronic hypophosphatemia, as phosphate is essential for energy metabolism, neuromuscular function, skeletal integrity, and numerous cellular processes [11]. Although phosphate and calcitriol supplementation constitute the primary medical therapy for unresectable or nonlocalized tumors and may provide temporary symptomatic improvement, their effectiveness in normalizing phosphate levels is often limited [2,12]. Consequently, despite preoperative oral supplementation, many patients fail to achieve adequate phosphate repletion and undergo surgery with low serum phosphorus concentrations [12]. Anesthesiologists should assess the severity of hypophosphatemia preoperatively and monitor serum phosphate when clinically indicated.

During the anesthetic planning and management of patients with TIO, particular attention must be given to their increased skeletal fragility and heightened risk of pathologic fractures. Careful patient positioning with meticulous padding of pressure points is essential to minimize the risk of injury [12]. Accordingly, in our patient, additional protective measures were implemented, including the use of foam padding and strategically placed blankets over vulnerable anatomical areas, particularly the hips and lumbar and cervical spine, to minimize mechanical stress and reduce the risk of positioning-related complications.

In patients with TIO, given the increased risk of iatrogenic fractures [13], careful handling during tracheal intubation is particularly important to reduce the risk of injury associated with cervical spine manipulation. Accordingly, our anesthesiology team utilized a video laryngoscope to facilitate intubation while minimizing cervical movement. Previous studies have demonstrated that video laryngoscopy is associated with higher first-attempt intubation success rates compared with direct laryngoscopy [14]. The video laryngoscopy technique may be particularly advantageous in patients with OSA and other anticipated difficult airway features due to its ability to improve glottic visualization and decrease airway manipulation [8]. Therefore, in our patient, the selection of this intubation technique was based on the goal of preserving cervical spine safety while ensuring a secure airway.

Considering the patient’s history of TIO-associated hypophosphatemia, hypertension, OSA, and reduced oxygen saturation on presentation, general anesthesia with endotracheal intubation was selected as the anesthetic technique. Although regional and local anesthetic approaches have been used successfully in patients with OSA [15], general anesthesia was deemed most appropriate in this case to ensure a secure airway, facilitate controlled ventilation, and allow for continuous physiologic monitoring and individualized hemodynamic management throughout the procedure.

Ventilatory management was also carefully planned to minimize factors that could further worsen preexisting hypophosphatemia. Rapid-sequence induction was therefore selected over standard induction to establish a secure airway while minimizing prolonged manual ventilation, which, if excessive, may result in respiratory alkalosis, promote intracellular phosphate shifts, and worsen hypophosphatemia [12]. Hypophosphatemia has been associated with impaired neuromuscular function and prolonged neuromuscular blockade secondary to reduced skeletal muscle ATP synthesis [11]. In addition, because patients with OSA are at increased risk of residual neuromuscular blockade and postoperative respiratory complications [16], minimizing overall exposure to nondepolarizing neuromuscular blocking agents was considered particularly important. Moreover, the patient’s severe osteomalacia and extensive history of pathologic fractures required an induction strategy that minimized the risk of musculoskeletal injury associated with succinylcholine-induced muscle fasciculations. Thus, a low dose of rocuronium was administered before succinylcholine as a defasciculating dose to mitigate this response [17]. Together, these considerations supported an individualized induction strategy designed to limit exposure to nondepolarizing neuromuscular blocking agents while optimizing conditions for emergence, extubation, and postoperative respiratory function.

Although a brief period of hypotension following induction with agents such as propofol and neuromuscular blocking agents is expected [18], our patient experienced a prolonged hypotensive episode that required vasopressor support until hemodynamic stability was achieved. The etiology of the hypotensive episode was likely multifactorial, resulting from a combination of anesthetic-induced vasodilation, advanced age, underlying physiologic frailty, and other cardiovascular-related effects. Although total IV anesthesia (TIVA) could have been considered for several reasons, including neurophysiologic monitoring, intracranial pressure considerations, rapid emergence, and hypertensive patients requiring strict BP control [19], the sustained hypotension observed after induction led to the use of inhaled anesthetic agents for maintenance of anesthesia because of their rapid titratability. Both volatile anesthesia and TIVA offer distinct advantages and limitations, but in certain clinical settings, volatile agents have been associated with shorter extubation times and may promote earlier recovery of spontaneous ventilation [20]. Despite concerns regarding impaired neuromuscular function associated with hypophosphatemia and OSA, the patient demonstrated adequate postoperative recovery without evidence of residual or prolonged neuromuscular blockade. The absence of quantitative neuromuscular monitoring, however, represents an important limitation, as confirmation of a train-of-four ratio ≥0.9 would have provided a more reliable assessment of neuromuscular recovery before extubation.

## Conclusions

This case highlights important anesthetic considerations in a patient with TIO secondary to an FGF23-secreting PMT. Chronic hypophosphatemia, skeletal fragility, potential respiratory muscle dysfunction, and coexisting conditions may present important perioperative challenges. In this patient, video laryngoscopy, rapid-sequence induction, limited use of neuromuscular blocking agents, and volatile anesthesia were successfully used as part of the individualized anesthetic management. However, the effectiveness or superiority of these strategies cannot be established from a single case.

Although TIO and severe hypophosphatemia may theoretically increase the risk of neuromuscular weakness, respiratory compromise, impaired recovery from neuromuscular blockade, and hemodynamic disturbances, none of these complications were observed in our patient. Postoperative respiratory monitoring, particularly in patients with coexisting OSA, as well as reassessment of serum phosphate and other relevant metabolic parameters, may be important considerations. Overall, this case emphasizes the importance of individualized perioperative assessment and multidisciplinary planning when managing patients with TIO and associated metabolic and skeletal abnormalities.
